# Facilitating an Interprofessional Course on Climate Change and Public Health Preparedness

**DOI:** 10.3390/ijerph20105885

**Published:** 2023-05-19

**Authors:** Heidi Honegger Rogers, Megan Tucker, Mary Pat Couig, Vanessa Svihla

**Affiliations:** 1 Office of Interprofessional Education, University of New Mexico Health Sciences, Albuquerque, NM 87131, USA; 2 College of Nursing, University of New Mexico, Albuquerque, NM 87131, USA; 3 Organization, Information & Learning Sciences, University of New Mexico, Albuquerque, NM 87131, USA; 4 Chemical & Biological Engineering, University of New Mexico, Albuquerque, NM 87131, USA

**Keywords:** climate change, health education, interprofessional education, action competence framework, interest development theory, public health preparedness

## Abstract

In this paper, we share the theories that guided the design of an interprofessional education course on *Climate Change and Public Health Preparedness* and how the course supported students’ professional interest and action competence as they move through their education and into their professional work in the context of our unfolding climate crisis. The course was guided by the public health emergency preparedness domains and was built to allow for students to explore applications of the content for themselves and their own profession. We designed the learning activities to support personal and professional interest development and help students move into perceived and demonstrated action competence. For the evaluation of our course, we asked the following research questions: What kinds of personal and professional commitments to action did students propose by the end of the course? Did these vary in depth and specificity and by the number of credits they enrolled in? In what ways did students develop personal and professional action competence over the course? Finally, how did they show personal, professional, and collective agency related to the course content on adaptation, preparedness, and mitigation of the health impacts from climate change? Using qualitative analysis guided by action competence and interest development theories, we coded student writing from course assignments. We also conducted comparative statistical analysis to assess differential impacts for students who enrolled for one versus three credits. The results show that this course design supported students’ progression of knowledge and perceived ability in specific individual and professional collective actions to reduce the health impacts of climate change.

## 1. Introduction

There is a growing need to frame climate change as a health issue [1,2] and to educate health and pre-health professionals to respond to this crisis as it is unfolding [3]. Climate change is a complex problem that requires interdisciplinary partnership [4]. It is also a topic that deserves attention and action, but research suggests that deep understanding of climate change can foster a sense of helplessness, which diminishes individuals’ capacity to act [5,6]. Therefore, education should center on capacity to act. Scholars have argued that climate change education for health professionals should be presented in an essential services of public health framework [7], but to date, few studies report on impacts of doing so in ways that also integrate learning theory that emphasizes capacity to act.

We share the design of a variable credit, graduate/undergraduate 8-week, online asynchronous course for health and pre-health professionals, the course content is available under Supplementary Materials. The course deliberately integrates the public health emergency preparedness domains [1], which are important aspects of the essential services of public health [7] with two pedagogical frameworks: interest development theory [8] and the action competence framework [9]. We share outcomes from this design-based research study to illustrate student progress related to this approach.

### 1.1. Pedagogical Frameworks

The course was organized around the public health preparedness model to promote resilience [1,10]. The course emphasized the four domains of public health emergency preparedness “… to prevent, protect against, quickly respond to, and recover from health emergencies, …” [1]. This framework helped to organize our modules into four foci: (1) climate change prediction, awareness, and preparedness; (2) health impacts with an adaptation and mitigation component; (3) public health infrastructure/disaster preparedness response and delivery systems; and (4) resilience and recovery. Yet, this comprehensiveness highlights a pedagogical challenge, that of the mile-wide, inch-deep curriculum. To avoid an overly packed course that would result in little learning, we turned to theories that offer guidance about how students develop interest and commitment to act. We used the interest development theory [8] and the action competence framework [9] as guidance for structuring the learning experiences. 

The interest development model is an empirically derived four-phase model that characterizes how learners engage with new content and suggests ways to help them make new learning meaningful through a process of exploration [8]. Phase 1 is triggered situational interest, where students have an opportunity to be surprised and to discover that new information has personal meaning and context. Phase 2 is maintained situational interest, where students are supported to apply new information to an ongoing personal or professional process. Phase 3 is emerging individual interest, where students choose to seek further information and meaning/application of the content. Phase 4 is well developed individual interest, where students perceive value in continued engagement with the material and incorporate the material into their personal and/or professional identity. This framework has been used as a central tenet in Science, Technology, Engineering, and Mathematics (STEM) education and helps to guide faculty to create learning experiences that are meaningful and spark long term engagement in subject areas [11]. 

Yet, developing health professions students’ interest in climate change and public health is not sufficient for changing personal and professional practices. To address this limitation, we sought guidance from the action competence framework—a key concept in environmental education for years, introduced to address concerns that typical approaches to teaching produced rather inert knowledge [9,12]. In this setting, action competence was initially defined as the ability to act on environmental concerns [9]. Since then, scholars distinguished action competence as an “ideal” rather than as basic behaviors learners can simply be trained to carry out, a differentiation that is necessitated by the complex interdisciplinary knowledge as well as problem framing and solving capacities needed to act in consequential ways [13]. Students skilled in action competence exhibit “commitment; willingness and courage to act; knowledge about consequences of and root causes to problems; knowledge about and a capability to develop visions and possible solutions to a problem; knowledge about how to influence and change conditions; and, finally, [ability] to put this knowledge into practice” [14]. 

In a recent systematic review on action competence, covering literature from 1997 to 2017, only 34 empirical studies were identified as focusing on educators’ use of action competence in a formal or informal educational context [12]. Of the 34 articles, 23 studies centered on an interdisciplinary project, the other 13 focused on science, art, social studies, geography, and education. These results highlight the importance of interdisciplinary climate change topics to create action competence, which has also been supported in other literature [15]. The majority (56%) of the review articles included were qualitative in design, allowing for a focus on the learning process as a whole [12]. Adopting an action competence approach to learning not only allowed for students to be aware of the issues surrounding climate change but helped to raise awareness of the problems and solutions. Student empowerment came from moving from individual ownership of the problems and solutions to thinking of how the collective (in a democratic society) could benefit [12]. Several studies published since this review highlight action competence development in classroom and informal settings. Youth programs provided participants the opportunity to move from personal sustainability-related actions to effecting change in their communities [16,17]. One longitudinal study in an educational environment found that action competence growth requires formal and ongoing Education for Sustainable Development (ESD) [18]. 

This definitional work illuminates that educating students toward the ideal of action competence depends on specific pedagogical designs. The action competence framework emphasizes the following: while learners may engage in projects that address climate change, the primary purpose should be in supporting their learning; and climate change is not just scientific, but also political, and therefore, learning should be participatory, intentional, and democratic [9,19]. In extending this, scholars have argued that supporting development of action competence entails “social learning, systemic and critical thinking, understanding of the complexity and interdisciplinary nature of real-life sustainability problems and their causes, envisioning a sustainable future, and the study of action possibilities for promoting real change” [20]. This approach helps learners move from being uninformed, in denial, or paralyzed from anxiety about climate change to a place of understanding and active engagement [21,22].

Across these pedagogical frameworks, we envision a trajectory from initial, situational interest through action competence. Interest can provide a solid foundation for further learning and growth [23]; this is in part because initial interest development intertwines with identity formation, shaping how relevant students may view the topics they are learning [24]. Yet, understanding how to bring this approach into health and pre-health professionals remains understudied. Within this domain, they must have skills in communication, interdisciplinary collaboration, mitigation, and solutions-focused adaptation methods that can be locally applied [25].

**Figure 1:**
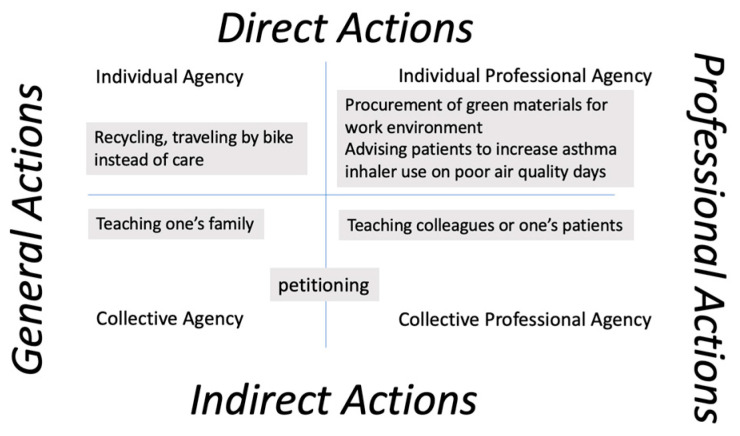
The public health context provides guidance on professional actions; intersected with an understanding of individual versus collective agency, we focus our efforts for developing interest and action competence on the collective professional agency quadrant.

To situate this work in the health professions, we foreground the role of agency, which has previously been invoked in action competence research [26,27], in part because perceived lack of agency can be a barrier to taking actions [28]. Recent research in this area has helped differentiate between individual and collective agency [29]. Intersecting this with the public health emergency preparedness domains [1], we can differentiate between direct versus indirect actions [9,30] and general versus professional actions (Figure 1). This helps clarify the need to support interest development toward professional, collective agency and action.

### 1.2. Learning Environment and Course Design

In designing the course, we sought to help students engage with the field of public health through the concepts of preparedness, mitigation, response, and resilience and recovery to understand how climate change needs to be thought of as a disaster and how professionals and communities can work together to reduce the worst possible outcomes. These public health emergency preparedness domains [1] provided an organized set of themes that we used to develop course modules. 

Students came into the course with varying levels of knowledge about climate change, health, and public health. We assumed most students who chose to take this elective course had already identified climate change as a topic they had personal interest in. Yet, we realized that while personal interest could serve as a motivating foundation, we primarily sought to develop their interest in climate change as health professionals. 

We promoted interest development [8] in a few ways. First, we supported triggered situational professional interest by providing resources and specific prompts to scaffold students to engage with new information while linking it to their communities, personal experiences, existing knowledge, and professions. For instance, as the course was taught during the COVID-19 pandemic—widely interpreted to be a consequence of a changing climate [31], we supported students to engage with this connection. Since each student came to the course from a different professional field and varying knowledge of how climate change works, we started by asking them to find new insights—or surprises—as a prompt to explore material they were not previously familiar with. This helped the instructors understand where students were starting. This also set the expectations that each student was going to be encouraged to engage with information they found interesting and applicable to their own professional development. Across modules, we continued this approach of introducing resources and engaging students in making personal and professional connections to maintain situational interest. For instance, in the second module, students chose a health impact and compared how it affected two different communities that they were familiar with. This allowed students to investigate climate change health impacts in places that had meaning for them to further develop situational interest.

To support emerging professional interests, we created tasks that helped students make connections to their future professional roles. We believed that by emphasizing choice, application, and future professional roles, we could deepen connection with the material and extend the impact of the course. Prompts focused on the student’s professional roles and asked students to envision their own futures, applying information from the course. For instance, in the fourth module, students explored the deep adaptation agenda [32] and discussed what they need to do to promote sustainability and resilience for themselves, their family, and community. They then set intentions for how they will integrate the material from this course into their personal and professional lives.

In addition, we used several strategies to support the development of action competence. We asked discussion questions that pulled in individual experiences and professional perspectives and encouraged interdisciplinary discussions on collaborative ways to address problems, enhancing social learning. We provided resources that helped represent the complex interplay of scientific, social, and economic factors, and prompted students to distinguish, classify, or evaluate the information they were learning. To make the material more applicable and personal we also engaged students in discussions around the concept of climate change as a slowly unfolding disaster, with identifiable impacts and specific needs in New Mexico. We also connected students to their own community, tasking them with exploring local initiatives and making commitments to take part in climate actions on our campus. We also centered issues and solutions specifically for New Mexico and the southwest, to allow students to recognize how they have already been impacted by climate change and to explore locally meaningful solutions for their communities. We wanted students to find their way into the work of climate change action in adaptation, mitigation, and resilience recovery and to identify both professional and personal action measures they can take.

## 2. Materials and Methods

### 2.1. Study Design

We used design-based research (DBR), an education research method that aims to jointly develop theory about learning and test designs for learning [33,34,35]. In contrast to laboratory-based studies and randomized, controlled trials, DBR studies are conducted iteratively, under real-world conditions, often by collaborative teams of instructors, learning scientists, and learning designers, as is the case in this study. Rather than aiming to produce generalizable knowledge, DBR studies develop transferable designs paired with contextual theory about how to support specific learning aims. While other research methods focus on contrasting *whether* one intervention is better than another, this educational research method is used widely in the learning sciences and valued for its capacity to provide insight into the conditions under which an intervention supports meaningful engagement by learners [33,34,35]. This insight supports later scalability of interventions and is a critical pivot point between early foundational and exploratory studies, and later multi-site efficacy studies [36,37]. Since its development in the 1980s and 1990s [33], DBR has been subject to rigorous and careful examination and revision to ensure that it can serve in this role effectively [34,38,39,40]. This effort has included defining standards for DBR. The first standard is that DBR is conducted as a collaboration between learning scientists and instructors working in a specific context, providing opportunities for theory-based planning and reflection, as is the case in this study. The second standard is a focus on instantiating theory into an instructional design and testing it under real-world conditions, including in ways that place theory in harm’s way, meaning that the theory and design should be tested systematically in ways that probe the conditions under which the theory functions [35,41]. In the current study, the variable credit option was used to place theory in harm’s way: specifically, those enrolled for three credits received the full design, whereas those enrolled for just one credit did not. 

Broadly, our aims were to investigate how a course on climate change and public health could build interest and foster action competence in students from various health programs. More specifically, this study was guided by the following research questions:

RQ1: What kinds of actions did students propose by the end of the course? Did these vary in specificity and by the number of credits they enrolled in?

RQ2: How and in what ways did students develop action competence over the course, as demonstrated in course assignments? How did they show personal, professional, and collective agency related to climate change?

### 2.2. Data Collection and Setting

Following norms in DBR, we conducted a single-site study, deliberately varying aspects of the design within a context, a Climate Change and Public Health Preparedness course at a Hispanic-Serving Institution. This approach provides an opportunity for analytic focus on the design, and in the continuum from foundational and exploratory research to design and development research like ours, to scale-up and efficacy research, a single-site study is appropriate [37]. We collected data from three iterations of the Climate Change and Public Health Preparedness course in 2021. Of the 73 enrolled students, 29 signed up for one credit, 12 for two credits, and 32 for three credits, and 57 were undergraduates. Most students were in nursing and population health programs, though others, who identified as pre-health professional students, came from biochemistry, psychology, engineering, exercise science, and liberal arts. Of the enrolled students, 69 completed an ungraded end-of-course evaluation that posed the following questions:

- *Describe your knowledge of action possibilities that you can take to help improve human health in the context of climate change.*
- *Describe your own confidence in taking these actions as you develop professionally.*
- *Are you leaving the course with a commitment to act towards a sustainable future?*

We also recruited case study participants from these same iterations. Fourteen students agreed to participate. We excluded students who took the course for one credit, as they did not complete module reflections. We excluded students who were missing two or more reflections. Our final case study group was eleven students (spring n = 4; summer n = 5; fall n = 2). We selected data from course assignments, including students’ written reflections from each module and their final assignment, which included questions about their beliefs of themselves as (pre-)professionals and how they would apply information from the course to their professional lives. The module reflections covered topics including eco-anxiety, mitigation, and adaptation to reduce the health impacts of climate change, the roles of health professionals in climate change prevention, and the themes of resilience, relinquishment, restoration, and reconciliation (refer to Appendix A). The volume of data across these 11 participants, paired with the full set of end-of-course evaluations, provided a robust dataset for establishing saturation, suggesting sample adequacy [42]. 

### 2.3. Data Analysis

To analyze the data, our research team developed a coding scheme based on the action competence framework for our first cycle coding [43]. Students’ written work was divided into data units, limiting the unit to one observable action regarding climate change. Data not containing an observable action were not coded, and actions were categorized as specific and detailed or as vague and general. The team then coded actions in terms of agency and domain (Table 1). Initial coding of a small segment of the dataset was done by three members of the research team who resolved any ambiguities and came to consensus. One member then coded a subset of the data and brought questions to the full team to resolve. Some of the questions centered around whether to code a data unit, because many actions were so vague, they lost any meaning. One example of a reflection discussed by the team was “Therefore addressing the fundamental changes that drought will cause is necessary to mitigate other disasters down the line”. The team discussed the fact that ‘addressing fundamental changes’ is so vague, the action could be interpreted in too many ways. The team agreed that a coded action must meet the threshold of something one could observe being enacted in the world. Once the coding scheme was finalized, two members of the team coded subsets of the data separately to establish intercoder reliability, meeting to resolve any differences. The coders worked through three subsets until they came to 100% consensus. Once they reached full consensus, the two coders divided the remaining data, with one coding 80% of the set and the other coding 20%.

For the second cycle coding, the research team synthesized our first cycle work using pattern coding [43]. Pattern coding afforded the research team the opportunity to pool codes across the data and attribute meaning to the results [43]. The team investigated comparisons across the student learning trajectories and across the coding categories. In analyzing relationships between codes, we looked for evidence of the degree to which the students have the capacity to act, and whether that capacity extended to their professional agency in the health domain. 

For the whole class data, which was also coded using the specificity and domain codes, we calculated descriptive statistics and used a version of the Chi square test appropriate to our sample, which was stratified by the number of credits each student enrolled in, using the Mantel–Haenszel test [44]. Because so few enrolled for two credits, we compared those enrolled for one versus three credits (n = 58). Chi square is an appropriate test because it can be used even with sample sizes smaller than 50 [45]. 

## 3. Results

We share results organized by research question. Our first question investigated the kinds of actions—personal, non-health-related professional, and health-related professional—students proposed on an end-of-course, ungraded assessment. We investigated whether there were differences between those enrolled for one versus three credits, conjecturing that the former likely displayed professional interest development, but the latter would also show development of action competence. 

We found that most students (70%) proposed a personal action and most of those (79%) were specific. Approximately one third (30%) proposed some other professional action, and almost one third (29%) of these were specific. Further, 58% offered a professional health related action, and just over half (55%) of those were specific. Looking across categories, only three students offered no specific or vague actions, yet these students tended to express that they gained knowledge, commitment, or awareness. This may align with research showing that students are not good judges of their capacities, especially at an early state [35], however, we also cannot rule out that, because these data were not tied to a course grade, some students simply put less effort in. 

**Figure 2:**
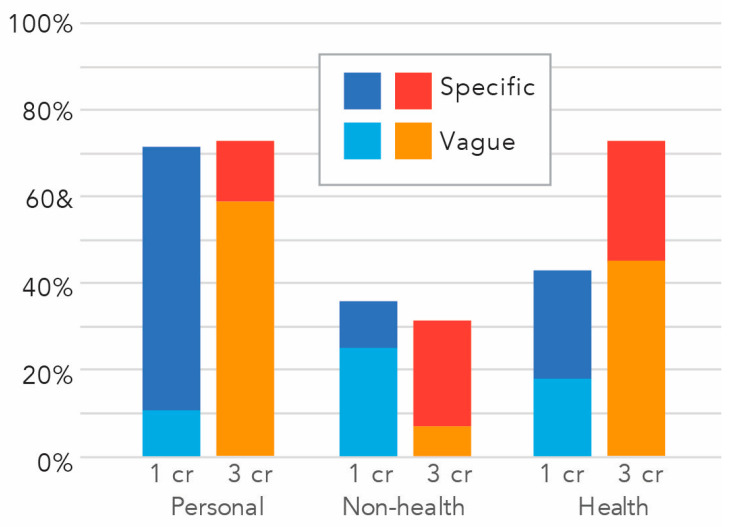
Percent of students proposing personal, non-health-related, and health-related actions by number of credits.

When we compared students enrolled for one versus three credits, we found similarities and differences. First, in terms of proposing personal and non-health-related actions, these groups were not statistically different, χ^2^(1, 58) = 0.05, *p* = 0.82, χ^2^(1, 58) = 0.33, *p* = 0.57, respectively. However, those enrolled for three credits were significantly more likely to propose a health-related action, χ^2^(1, 58) = 3.83, *p* < 0.05 (Figure 2).

Our second research question investigated ways case study students developed professional interest and action competence over the course, as demonstrated in course assignments. We wondered how they showed individual and collective agency related to climate change.

**Figure 3:**
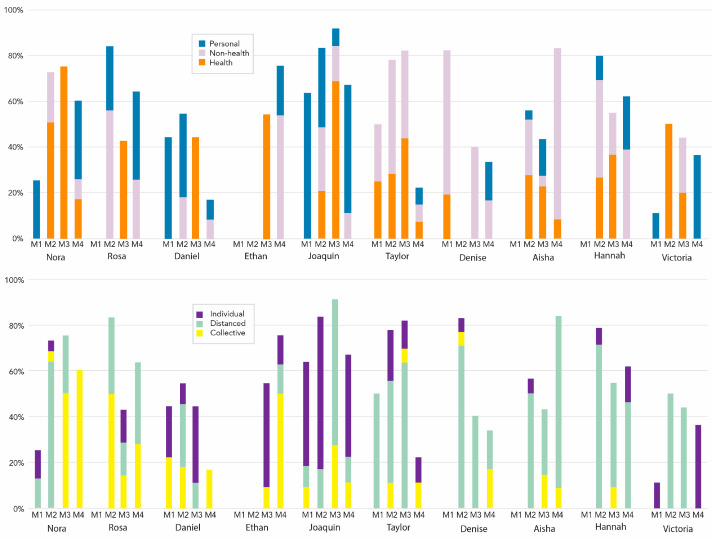
Case study students’ personal, non-health, and health-related actions, proposed as individual, distanced, or collective actions across four modules (M1–M4).

Across four module reflections, students varied in their proposals for health-related actions and in demonstrating collective agency (Figure 3). Only two (Taylor and Denise—names were created to protect the identities of the study participants) mentioned health-related actions in their first reflection. One student, Amira, is omitted due to a missing reflection.

**Figure 4:**
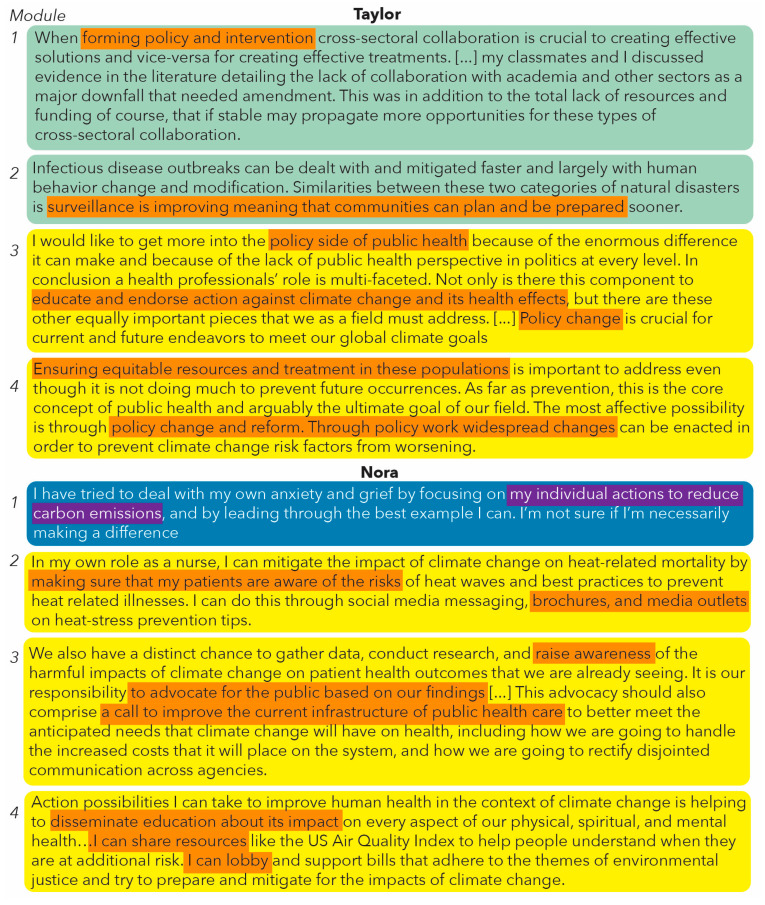
Examples from two case study students’ reflections across modules; color scheme as defined in Figure 3.

We selected two students to illustrate different paths through the course: Taylor displayed initial interest in health and deepened this into action competence; Nora initially displayed personal action competence and developed both interest and health-related action competence. We detail their trajectories in turn (Figure 4).

Taylor, a graduate student taking the course for three credits, shared health-related action in their first reflection, though the action is vague and showed distanced agency. In their module two reflection, Taylor mostly identified vague actions, but did point to surveillance as a specific health-related action and suggested actions from a distanced point of view, rather than showing individual agency. In module three, Taylor continued to identify health-related actions, moving into an evaluation of the areas of action a health professional can take. Here, Taylor took up a collective, professional stance. For their final module four assignment, Taylor reflected on the goals of public health professionals, positioning themself as a member of the public health profession, showing collective agency. They showed evidence of the more-developed phases of the interest development framework, as their writing suggested they are re-engaging with ideas throughout the course and are reflective about the content. They recognized that indirect action, through policy change, will have a larger impact on the populations they will serve.

Nora, an undergraduate student taking the course for three credits, started the course focusing on personal actions. Nora reflected on the impact of climate change but was vague about how to counter the impact. In their module two reflection, Nora identified specific actions they can take as a health professional, in places, sharing care-related actions, but also considering how they could communicate with their patients regarding health consequences of climate change. In module three, Nora continued to focus on specific, health-related action. In this reflection, they took a collective stance. In the final course assignment, Nora shared specific, health-related actions they can take. Throughout the course, Nora showed a growing awareness of professional actions. In addition to growing their action competence, Nora showed evidence of the more-developed phases of the interest development framework, as they engaged with and reflected deepening knowledge of the course content.

## 4. Discussion and Limitations

Our primary aim was to investigate how a course on climate change and public health could build interest and foster action competence in students from various health programs by analyzing reflections written by students who enrolled in the course for one, two, or three credits. As only a small fraction of published papers in the area of climate change and health report on interventions [46], our study contributes to both research and practice. Specifically, with only five such papers using mixed methods, our study offers insight into how we can make climate change education accessible for health professionals, and it sheds light on the effects of an intervention on the development of students’ professional interest and capacity to act related to climate change. We discuss the course design in relation to our findings, their limitations, and ways future studies may address these.

First, in order to make the course accessible to a range of students, we designed it to meet elective credit needs for any graduate or undergraduate student at the University of New Mexico. We were successful in attracting both graduate and undergraduates from various disciplines, and this led to interesting conversations on a wide variety of perspectives of module content. We introduced the students to a public health emergency response framework to increase their interest in the topic of climate change and health and to give them a learning space to explore how they can apply information from this field into their own personal and professional domains. Future courses could further integrate the essential core functions of public health [7] to allow students to further understand the field of public health systems and integrate content into actions. 

Second, we sought to investigate the kinds of actions students proposed and whether this varied by the number of credits they enrolled in. The variable credit design jointly encouraged students to enroll and allowed those enrolled for more credits to deepen their exploration and application of the content. The variable credit option fostered interest development, even for those enrolled for one credit. While these students offered less specific professional actions compared to their peers who enrolled for three credits, their increased interest may serve as a foundation for further learning, as others have argued [23]. One limitation relates to our use of a Chi square test. Although our data met the Chi square assumptions and our sample size of 58 met norms [45], there is a risk that we lacked adequate power, resulting in a Type II error (as this test is robust even with small sample sizes to Type I errors [45], meaning that there may have been other, more subtle differences that we failed to detect. Another limitation of the current study is that we did not follow students after the course ended to investigate whether they acted on this interest or took the actions they described. In particular, we wonder how those who enrolled for different credits sought out new opportunities to learn and to take action. Future studies may remedy this via follow-up interviews or surveys with participants, offering an opportunity to relate perceived and actual professional action competence. In this vein, our results also align with research showing that short-duration courses seldom reach the highest levels of well-developed interest [47], which in turn suggests the need for studies that span multiple related courses in a program.

Third, our approach to action competence distinguished between every day, personal actions that students likely had foreknowledge of and professional actions that they likely learned about in the course. However, our study did not focus on conceptual gains related to the course content. Future studies can address this limitation by adapting existing, research-based assessments from adjacent domains and deploying a pre/post assessment design, by using pre-assessments to investigate students’ familiarity with topics in course readings, and by investigating the cumulative retention of course concepts in a follow-up course. In addition, as the field has clarified the importance of taking a systems approach [48], new assessments are needed to trace the development, not only of conceptual knowledge, but of students’ developing capacity to represent and address problems as complex systems.

Fourth, design-based research (DBR), our methodological approach, provided an opportunity to test a specific instructional design under particular conditions, leading to insights about how the design functioned. DBR is valued for its focus on deliberately instantiating theories of learning and testing them under real-world conditions [34,35], and often in ways that rely on existing sources of data, such as student work and evidence of interactions during learning. In contrast to well-controlled laboratory-based studies, DBR studies tend to produce results that are more transferrable to other authentic classroom contexts. In our study, the variable credit provided an endemic basis of comparison, allowing us to test variants of our theory synchronously, resulting in understanding of the impact of variable credits on the development of interest and action competence detailed above. However, there are two limitations that must be considered. First, as commonly practiced, DBR focuses on gathering process data, such as video recordings of students interacting during class sessions. With a fully online, asynchronous class, we were limited to written interactions, and this presents several possible issues: Students’ writing is likely more formal than their face-to-face (or video conference) conversations would have been, and they may have relied on resources in crafting written statements, which were graded, producing writing that answered the prompts but that may have masked their underlying values. This is one reason DBR studies tend to incorporate video data, which offers more ways to interpret the learning process. Future studies can address this limitation by contrasting different versions of the course, including those taught face-to-face and those requiring synchronous video conferencing, to investigate how students gain perspectives from one another and how such interdisciplinary collaborations might foster interest development and professional action competence. Second, methodologically, DBR does not produce generalizable results, instead focusing on transferability [34]. While multi-site studies are laudable, policies on education research attest to the value that studies like ours can bring as a precursor to such studies [37], offering deeper understanding of contextual variance and conditions that matter in the intervention; this is in part because even multi-site, randomized, controlled designs may fail to predict impacts at new sites if they differ from those in the original study set [49]. Our particular context—a Hispanic serving research university, and historical moment—during a pandemic, may have shaped students’ participation and learning in systematic ways. For instance, our students may bring their experiences of interacting in our diverse community and courses to bear as they consider health disparities in ways that those enrolled in predominantly white institutions might not. Certainly, students cited the pandemic as a motivating factor in their desire to understand climate change and health. Additional research contrasting similar courses taught at different institutions can provide insight into the generality of our results. However, we argue that explicit discussion of the learning theory undergirding the design is instrumental to such development. In other words, we would not predict that a course covering the same content—but taught using lectures and quizzes—would have the same impacts on interest and action competence development because of the known complexity of these learning outcomes [9,19].

## 5. Conclusions

We designed a course to optimize the interprofessional nature of student enrollment, to enhance interest development in the field of climate change and health, and to foster personal and professional action competence for climate change mitigation. The course was designed with three frameworks to support the development of interest and action competence for working in the field of climate change and health, public health preparedness, and climate change and health communications. The design maximizes flexibility by being offered as variable credit and both undergraduate/graduate credit. We studied the impact of the course over three offerings, finding students developed professional interest and capacity to commit to personal and professional actions, though those enrolled for three credits were significantly more likely to propose health-related actions. Our results suggest there is value for a variable credit approach in terms of maximizing access but also demonstrate that students benefit from more intensive experiences. Our results also foreground the value of developing courses informed by frameworks that guide practice, like the public health emergency preparedness domains [1] and theories of learning, such as interest development [8] and action competence [9]. Given the complexity and interdisciplinarity of climate change impacts on health, integrating these frameworks was critical to our success.

## Figures and Tables

**Table 1:** First cycle coding scheme. Student work was coded first for vague and specific actions; next, we assessed the agency and domain of the action.

Code	Description	Example
**Agency**		
**Individual**	Action tied to “I”	“I’ve tried my best to reduce carbon emissions”
**Collective**	Action tied to “We”, “Our”, or “Us”	“Also, as nurses when we speak, legislators listen. We are the ones watching and researching impacts we see and experience in our communities.”
**Distancing**	Action tied to generic “You” or “One”	“Educating students in schools can be an effective way of motivating the next generations to bring about positive changes.”
**Domain**		
**Personal**	Action can be completed by any individual	“We could let go of our reliance on single-use plastics and promote alternatives that are designed to break down”
**Health** **related**	Action or profession is related to health	“I can assist in educating other healthcare professionals, the community and patients that I see in clinic on broad ideas surrounding health effects of climate change as well as health conditions that we will see more of in this area of the country.”
**Non-health-related/professional**	An action or profession that is related to a professional domain outside of health	“Carbon trading means that emissions limits are set for companies and if they go beyond those limits, they must pay, but if they go under those limits, they can receive cash.”
**Interest development related to health-related climate actions**		
**Situational interest**	Engagement is externally supported, without expression of interest, curiosity, or self-directed planned learning	“One health impact of climate change is that it can lead to water-borne diseases and other water-related health issues (WHO, 2021).”
**Emerging professional interest**	Engagement includes expression of interest or curiosity, evaluation of actions as important, self-directed plans for further learning	“I have started building knowledge regarding health implications of climate change as a result of the reading and research that I’ve done in this class, but feel I still have far to go.”

## Data Availability

Data for this study can be requested by contacting the corresponding author.

## Supplementary Materials

https://digitalrepository.unm.edu/hsc_ipe_fr/1/.

## Appendix A

**Table A1:** Module prompts.

Module	Reflection Prompts
1	Discuss the Wikipedia (community-built) page on the Psychological Impacts of Climate Change, what information resonates with you? How have you been able to manage (or not manage) your own worry, anxiety, or grief about climate change? How has your experience with the COVID-19 Pandemic raised your awareness of how human health is informed by the health of our environment (so far)? For ideas here, you can explore the Planetary Health Alliance.
2	In this reflective journal assignment, you will be exploring the concepts of mitigation and adaptation to reduce the health impacts of climate change in the US. Pick 2 or 3 different health impacts and discuss the work of health, public health, building and planning, policy, program, educational or community interventions that can be implemented. Discuss your own role in your chosen field and how you can be useful in this work to mitigate the health impacts of climate change in your own community. You may find some good resources in the Lancet Countdown US Policy Brief.
3	If you are a health professional or pre-health professional student, please explore the article on the critical roles of health professionals in climate change prevention and preparedness and write an essay on this topic from your own perspective. Where do you see health professionals in this work, what is your experience in your own community with people working on prevention and preparedness and what else can people in the health field do to support their communities? If you identify as a professional in a different field, please look for information and resources on how your profession plays a role in reducing the impacts of climate change on community/ecological health and/or well-being and discuss this. Explore the resources on the international COVID-19 response and give your ideas/thoughts/insights into the essential functions of government funded public health responders in preventing, mitigating and organizing health systems responses to events like this in the future.
4	Explore the work by Jem Bendell and respond to the 4 questions of the Deep Adaptation Framework for yourself: Resilience: what do we most value that we want to keep and how? Relinquishment: what do we need to let go of so as not to make matters worse? Restoration: what could we bring back to help us with these difficult times? Reconciliation: with what and whom shall we make peace as we awaken to our mutual mortality?
